# Gain of Chromosome 6p Correlates with Severe Anaplasia, Cellular Hyperchromasia, and Extraocular Spread of Retinoblastoma

**DOI:** 10.1016/j.xops.2021.100089

**Published:** 2021-12-11

**Authors:** Gustav Stålhammar, Aaron Yeung, Pia Mendoza, Sander R. Dubovy, J. William Harbour, Hans E. Grossniklaus

**Affiliations:** 1 Ocular Pathology Service, St. Erik Eye Hospital, Stockholm, Sweden; 2 Department of Clinical Neuroscience, Karolinska Institutet, Stockholm, Sweden; 3 Royal Victorian Eye and Ear Hospital, Melbourne, Australia; 4 Departments of Ophthalmology and Pathology, Emory University School of Medicine, Atlanta, Georgia; 5 Department of Ophthalmology, Bascom Palmer Eye Institute, University of Miami Miller School of Medicine, Miami, Florida; 6 Sylvester Comprehensive Cancer Center, University of Miami Miller School of Medicine, Miami, Florida; 7 Interdisciplinary Stem Cell Institute, University of Miami Miller School of Medicine, Miami, Florida

**Keywords:** Anaplasia, Chromosome 6p, Retinoblastoma, Survival, Whole genome sequencing, AJCC, American Joint Committee on Cancer, FFPE, formalin-fixed and paraffin-embedded, FISH, fluorescence in situ hybridization, IIRC, International Intraocular Retinoblastoma Classification, *NOL7*, nuclear protein 7, OD, optical density, SD, standard deviation

## Abstract

**Purpose:**

Gain of chromosome 6p has been associated with poor ocular survival in retinoblastoma and histopathologic grading of anaplasia with increased risk of metastatic spread and death. This study examined the correlation between these factors and other chromosomal abnormalities as well as results of whole genome sequencing, digital morphometry, and progression-free survival.

**Design:**

Retrospective cohort study from 2 United States tertiary referral centers.

**Participants:**

Forty-two children who had undergone enucleation for retinoblastoma from January 2000 through December 2017.

**Methods:**

Status of chromosomes 6p, 1q, 9q, and 16q was evaluated with fluorescence in situ hybridization, the degree of anaplasia and presence of histologic high-risk features were assessed by ocular pathologists, digital morphometry was performed on scanned tumor slides, and whole genome sequencing was performed on a subset of tumors. Progression-free survival was defined as absence of distant or local metastases or tumor growth beyond the cut end of the optic nerve.

**Main Outcome Measures:**

Correlation between each of chromosomal abnormalities, anaplasia, morphometry and sequencing results, and survival.

**Results:**

Forty-one of 42 included patients underwent primary enucleation and 1 was treated first with intra-arterial chemotherapy. Seven tumors showed mild anaplasia, 19 showed moderate anaplasia, and 16 showed severe anaplasia. All tumors had gain of 1q, 18 tumors had gain of 6p, 6 tumors had gain of 9q, and 36 tumors had loss of 16q. Tumors with severe anaplasia were significantly more likely to harbor 6p gains than tumors with nonsevere anaplasia (*P* < 0.001). Further, the hematoxylin staining intensity was significantly greater and that of eosin staining significantly lower in tumors with severe anaplasia (*P* < 0.05). Neither severe anaplasia (*P* = 0.10) nor gain of 6p (*P* = 0.21) correlated with histologic high-risk features, and severe anaplasia did not correlate to *RB1*, *CREBBP*, *NSD1*, or *BCOR* mutations in a subset of 14 tumors (*P* > 0.5). Patients with gain of 6p showed significantly shorter progression-free survival (*P* = 0.03, Wilcoxon test).

**Conclusions:**

Gain of chromosome 6p emerges as a strong prognostic biomarker in retinoblastoma because it correlates with severe anaplasia, quantifiable changes in tumor cell staining characteristics, and extraocular spread.

Retinoblastoma is the most common intraocular malignancy in children, with a global incidence amounting to approximately 8000 new cases per year.^1^^,^^2^ Major therapeutic advantages, including systemic, intra-arterial, and intravitreal chemotherapy, and focal treatment alternatives such as transpupillary thermotherapy, cryotherapy, and plaque brachytherapy, have all greatly contributed to increasing survival as well as globe conservation rates.^2^^,^^3^ Enucleation is still performed in cases where the tumor cannot be controlled with eye-preserving treatment. The prediction of which eyes can avoid enucleation has commonly been based on the International Intraocular Retinoblastoma Classification (IIRC).^4^ The classification is predictive of treatment success in 50% of cases in advanced-stage disease, which indicates that early enucleation is a valid treatment option for the other half.^5^, ^6^, ^7^ More recently, a classification from the American Joint Committee on Cancer (AJCC) showed an even higher predictive value, with a 5-year globe-salvage rate of only 25% for cT3 tumors.^8^^,^^9^

Nonetheless, novel reliable indicators of aggressive disease are of great importance. We previously showed that histopathologic grading of severe anaplasia in retinoblastoma correlates with histologic high-risk features and is associated with increased risk of metastasis and decreased patient survival.^10^ In addition to *RB1* loss, gain of chromosome 6p has been found to be present in most patients with retinoblastoma.^11^ A distinct gene expression profile has been shown to distinguish between severe and nonsevere anaplasia, which included the *DEK* gene located on chromosome 6p22.3.^12^ Chromosome 6p gain often presents as isochromosome 6p and preferentially has been identified in poorly differentiated tumors with unfavorable clinical outcomes.^13^^,^^14^ Many chromosomal aberrations induce changes in the size and shape of cells, as well as in their staining and growth pattern. These changes can often be appreciated; for example, the degree of tumor differentiation in retinoblastoma is evaluated during the diagnostic workup by ophthalmic pathologists, but may be hard to quantify and reproduce between observers.^15^, ^16^, ^17^ Therefore, automated digital measurement of these features is an attractive alternative for the human eye.^18^, ^19^, ^20^, ^21^ Examinations of tumor-derived cell-free DNA and chromosomal copy-number alterations were recently shown to detect gain of 6p and a range of other chromosomal alterations in minute volumes of aqueous humor, including gain of 1q, loss of 16q, and focal *MYCN* amplificiation.^7^^,^^22^ Importantly, cell-free DNA amplification has been determined to be a prognostic biomarker of ocular survival in retinoblastoma in both aqueous humor samples obtained in vivo and from eye specimens after enucleation.^22^ However, gain of 6p remains to be correlated to systemic outcomes, including risk for extraocular spread and progression-free and disease-specific survival.

In this study, we examined the relationship between degree of anaplasia and a range of these factors, including chromosome 1q, 6p, 9q, and 16q status; automated digital measurement of 33 different tumor cell size and staining characteristics; mutations in *RB1*, *CREBBP*, *NSD1*, and *BCOR*; amplification of *MYCN*; and extraocular spread of retinoblastoma.

## Methods

### Tumor Samples

A total of 42 formalin-fixed and paraffin-embedded (FFPE) eyes from children who had undergone enucleation for retinoblastoma were included in this study, along with basic clinical information about patient age at enucleation, laterality, secondary tumors, development of metastases, and retinoblastoma-related death. Twenty-eight of these eyes were included from the archives of the Emory Eye Center, Atlanta, Georgia, and 14 from the archives of Bascom Palmer Eye Institute, Miami, Florida. Inclusion criteria were: enucleation from January 2000 through December 2017, availability of the eye in FFPE blocks, and access to survival data. Exclusion criteria were: less than 2 microscopic low-power fields (×20) of tumor tissue available (n = 1), extensive necrosis (n = 1), and diffuse tumor growth patterns (n = 0). All tumors were evaluated for the degree of anaplasia by ophthalmic pathologists and with fluorescence in situ hybridization probes for chromosomes 1q, 6p, 9q, and 16q. The tumors from Emory Eye Center also were assessed for histologic high-risk features and were analyzed with digital morphometry for the size and staining features of tumor cells, and the tumors from Bascom Palmer underwent whole genome sequencing, as described below. The protocol for data collection and analysis of specimens was approved by the Emory University Institutional Review Board (identifiers, 00028367 and 00069328). Informed consent was waived because no protected clinical information was collected and no interventions, treatments, meetings, or other contacts with patients or relatives were carried out. The study adhered to the United States Health Insurance Portability and Accountability Act and to the tenets of the Declaration of Helsinki of 1975, as revised in 1983.

### Histopathologic Evaluation

**Figure 1:**
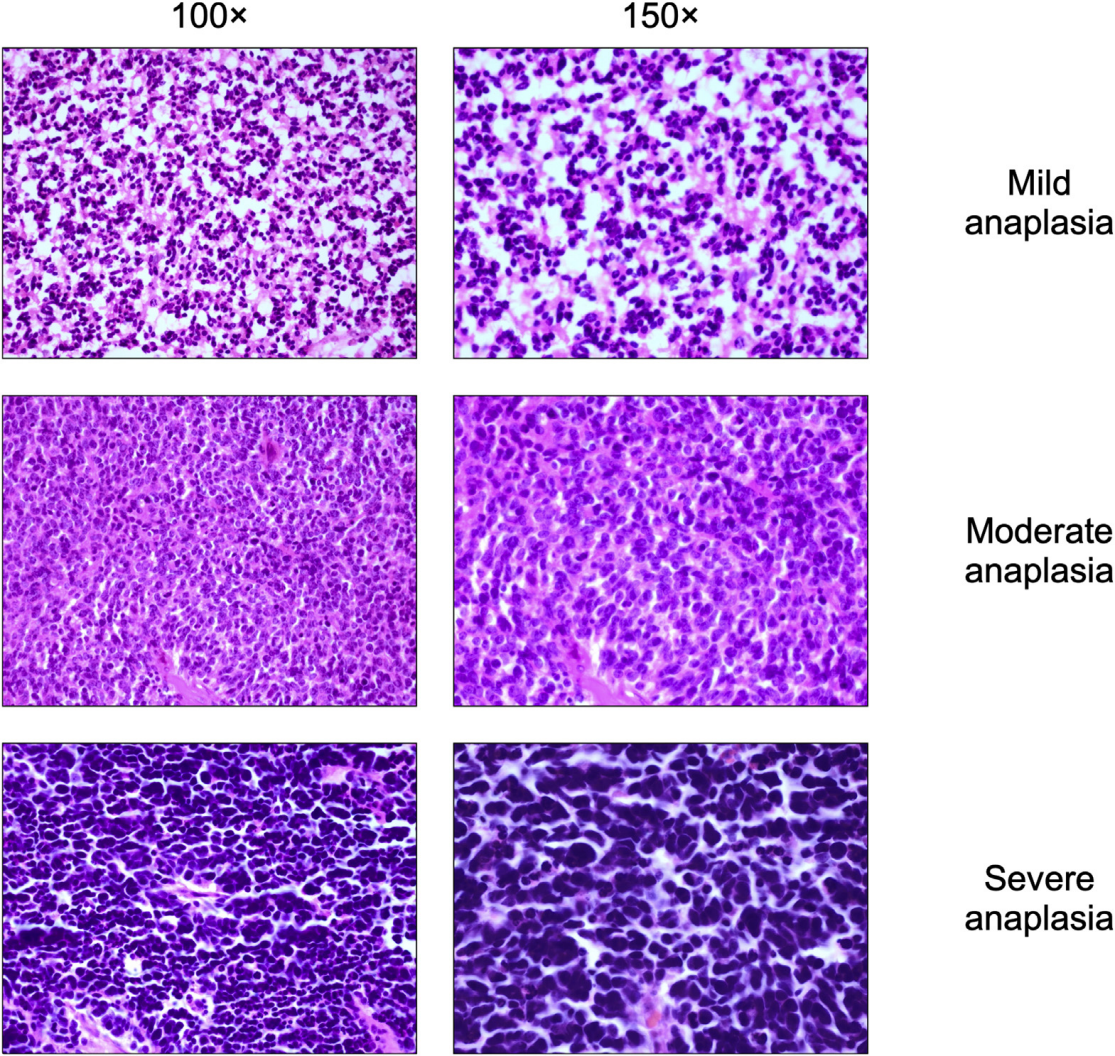
Photomicrographs showing examples of retinoblastomas with different degrees of anaplasia: (Top row) mild anaplasia, (Middle row) moderate anaplasia, and (Bottom row) severe anaplasia (original magnification, ×100 [left column] and ×150 [right column]; stain, hematoxylin–eosin).

One to 4 5-μm sections through the center of the pupil, tumor, and optic nerve of each eye were examined and graded according to International Retinoblastoma Staging Working Group and AJCC, 8th edition, recommendations by 3 ophthalmic pathologists (G.S., P.M., and H.E.G.).^23^^,^^24^ The histopathologic analysis in a light microscope (Olympus BHTU) included evaluation of tumor size (greatest basal dimension and thickness of tumor in millimeters), growth pattern (exophytic, endophytic, or combined), level of differentiation (undifferentiated, mild, or moderately or well differentiated as reflected by the presence or absence of fleurettes and Homer Wright and Flexner-Wintersteiner rosettes), tumor seeding (vitreous, subretinal, or both), extent of tissue invasion (anterior segment, choroid, extrascleral, or a combination thereof), extent of optic nerve invasion (prelaminar, laminar, postlaminar, or to the surgical margin of resection), degree of viable (intact) versus necrotic tumor, degree of apoptosis, degree of anaplasia (mild, moderate, or severe, Figure 1), and presence or absence of retinocytoma, all as described previously.^10^ The highest grade of anaplasia in 10% or more of the viable tumor was used to assign sample grade. Massive choroidal invasion was defined as full-thickness choroidal replacement with retinoblastoma in contact with 3 mm or more of contiguous sclera.

### Digital Cell Morphometry

Glass slides of tumor tissue stained with hematoxylin and eosin were digitally scanned at ×200 using the Hamamatsu Nanozoomer 2.0HT (Hamamatsu Photonics). The resulting images (resolution, 0.45 μm per pixel) were imported to the QuPath Bioimage analysis software version 0.2.0.^25^ The 3 ophthalmic pathologists (G.S., P.M., and H.E.G.) cooperated in consensus to define 2 circular regions per tumor that were representative of its degree of anaplasia. Each region had a diameter of 0.5 mm, which corresponds to the field of view in a light microscope with a ×400 objective and a combined area of 0.39 mm^2^ per tumor. Areas with intense inflammation, calcifications, bleeding, necrosis, tissue folds, or poor fixation were excluded from analysis. A workflow for morphometric analysis was then created, including the following steps for each tumor. First, calibration of the staining intensities of hematoxylin (cell nucleus) and eosin (cytoplasm) was carried out to adjust for the impact of any intertumor differences in staining intensity and nuance. Second, identification of all cells within the regions of interest was carried out using the software’s cell detection function with the following settings: background nucleus radius, 8 μm; median filter radius, 0 μm; sigma, 1 μm; minimum nucleus area, 5 μm^2^; maximum nucleus area, 50 μm^2^; threshold, 0.2; maximum background intensity, 2; and cell expansion, 4 μm. Third, measurement of 33 different parameters related to the shape, size, and staining patterns of each tumor cell nuclei and cytoplasm within the marked area was carried out according to a previously published method (Table 1).^18^ The image analysis software was run on a standard off-the-shelf laptop computer (Apple, Inc).

**Table 1:** Cell Morphometric Variables Analyzed

Variable	Description and Interpretation
Nucleus	
Area	Mean nucleus area (μm^2^)
Perimeter	Mean nucleus perimeter (μm)
Circularity	Mean nucleus circularity. Compares the perimeter of a shape with the area it contains. The circularity of a circle is 1.00, and less for less circular objects.
Maximum caliper	Mean nucleus length in longest dimension (μm)
Minimum caliper	Mean nucleus length in shortest dimension (μm)
Eccentricity	Mean nucleus eccentricity. A measure of how much the nucleus deviates from a spherical shape. A completely spherical nucleus has an eccentricity of 0.00, a nucleus with the shape of an elliptical 3D solid has an eccentricity of 0.5, whereas a 3D conical distribution has a value of 1.00.
Hematoxylin OD	
Mean	Mean nucleus hematoxylin stain intensity
Sum	Mean sum when the hematoxylin stain intensity of all pixels in a nucleus are added together
SD	SD of nucleus hematoxylin stain intensity
Maximum	Mean of strongest nucleus hematoxylin stain intensity
Minimum	Mean of weakest nucleus hematoxylin stain intensity
Range	Mean hematoxylin stain intensity range
Eosin OD	
Mean	Mean nucleus eosin stain intensity
Sum	Mean sum of eosin stain intensity when all pixels in a nucleus are added together
SD	SD of nucleus eosin stain intensity
Maximum	Mean of strongest nucleus eosin stain intensity
Minimum	Mean of weakest nucleus eosin stain intensity
Range	Mean eosin stain intensity range
Cell	
Area	Mean cell area (nucleus plus cytoplasm; μm^2^)
Perimeter	Mean cell (nucleus plus cytoplasm) perimeter (μm)
Circularity	Mean cell circularity (see definition of circularity above)
Caliper	
Maximum	Mean cell length in longest dimension (μm)
Minimum	Mean cell length in shortest dimension (μm)
Eccentricity	Mean cell eccentricity (see definition of circularity above)
Eosin OD	
Mean	Mean cell (nucleus plus cytoplasm) eosin stain intensity
SD	SD of cell (nucleus plus cytoplasm) eosin stain intensity
Maximum	Mean of strongest cell (nucleus plus cytoplasm) eosin stain intensity
Minimum	Mean of weakest cell (nucleus plus cytoplasm) eosin stain intensity
Cytoplasm hematoxylin OD	
Mean	Mean cell (nucleus plus cytoplasm) hematoxylin stain intensity
SD	SD of cell (nucleus plus cytoplasm) hematoxylin stain intensity
Maximum	Mean of strongest cell (nucleus plus cytoplasm) hematoxylin stain intensity
Minimum	Mean of weakest cell (nucleus plus cytoplasm) hematoxylin stain intensity
Nucleus-to-cell area ratio	Cell area divided by nucleus area

OD = optical density; SD = standard deviation; 3D = three-dimensional.

### Fluorescence In Situ Hybridization

Fluorescence in situ hybridization was performed on the FFPE retinoblastoma tumor tissue. The Vysis locus specific identifier DEK/NUP214 dual-color, dual-fusion translocation fluorescence in situ hybridization probe kit (Abbott Molecular Inc) was used according to the manufacturer’s protocol, using probes for chromosome regions 6p (DEK SpectrumGreen probe spanning chr6:17,754,135-18,705,577 on 6p22.3) and 9q (NUP214 SpectrumOrange probe spanning chr9:133,641,434-134,512,630 on 9q34.12-q34.13).^26^ Probes were viewed using a fluorescence microscope, allowing visualization of the orange and green fluorescent signals. For all cases, 100 cell nuclei were counted. Gains were defined as the detection of 2 or more signals in 1 cell nucleus. Information from whole genome sequencing was not added for the classifications.

### Whole Genome Sequencing

DNA was extracted from FFPE blocks using an 8-mm trephine and the QIAamp DNA Mini Kit (Qiagen). Matched tumor and healthy DNA samples were sequenced to ×30 depth for normal samples and at least ×200 depth for retinoblastoma samples using paired-end 150-bp reads. Reads were mapped to the hg38 reference genome using the Burrows-Wheeler Aligner Maximal Exact Match.^27^ Polymerase chain reaction duplicate reads were removed from the alignment using Picard tools (http://picard.sourceforge.net/). All germline variants were called using GATK4 Haplotype Caller and GenotypeGVCF walkers, whereas somatic variants were identified using GATK4 Mutect2, following GATK best practices.^28^, ^29^, ^30^ Variants were functionally annotated using single-nucleotide polymorphism effect (snpEff), and mutation significance covariates were used to identify significant somatic variants using variant allele frequency.^31^^,^^32^ Copy number segments were annotated to genes, and regions with empirically derived cutoffs were used to categorize a segment as deletion or amplification. Special attention was paid to the *MYCN*, *CREBBP*, *NSD1*, *BCOR*, and *RB1* genes, which are relevant to retinoblastoma.^33^, ^34^, ^35^

### Statistical Methods

Differences with a *P* value of < 0.05 were considered significant, all *P* values being 2-sided. The deviation from normal distribution of the following continuous variables were statistically significant when evaluated by the Shapiro-Wilk test (*P* < 0.05): nucleus area, nucleus hematoxylin optical density (OD) mean, nucleus hematoxylin OD standard deviation (SD), nucleus hematoxylin OD minimum, nucleus hematoxylin OD range, nucleus eosin OD SD, nucleus eosin OD range, cell area, cell circularity, cell eosin OD SD, cell eosin OD maximum, cytoplasm hematoxylin OD SD, cytoplasm hematoxylin OD maximum, and cytoplasm hematoxylin OD minimum. In tests of the null hypothesis in tumors with severe versus nonsevere anaplasia, these variables were evaluated with the Mann–Whitney *U* test. All other variables were evaluated with the Student *t* test. Contingency tables and the Fisher exact test were run to test the correlation between the following categorical variables: gain of 6p as determined by fluorescence in situ hybridization, severe anaplasia as determined by histopathologic examination, histologic high-risk features (defined as > 3 mm choroidal invasion, anterior segment invasion, postlaminar optic nerve invasion, or any degree of combined choroid and optic nerve invasion), and metastasis. For any significant differences, we calculated the relative risk for the genetic aberration if the degree of anaplasia was severe, according to the method described by Altman.^36^ For stepwise correlation among mild, moderate, and severe degrees of anaplasia and gain of 6p, linear regression was performed with the intercept included in the model. Progression-free survival was calculated with cumulative incidence analysis. Tumor progression was defined as presence of distant or local metastases or tumor growth beyond the cut end of the optic nerve. All statistical analyses were performed using IBM SPSS statistics version 25 (IBM).

## Results

### Descriptive Statistics

The average patient age at enucleation was 1.4 years (SD, 0.9 years). One of the 42 patients received other treatments before enucleation: a child with a 7 × 7 × 1-mm pT3b tumor with extensive subretinal and vitreous seeding. She received intra-arterial chemotherapy with melphalan, carboplatin, and topotecan and then underwent enucleation for poor response. On histologic examination, the eye was found to have postlaminar tumor growth. The other 41 patients underwent primary enucleation. Twenty-nine children had unilateral disease and 13 children had bilateral disease. Of 42 tumors, 7 had mild anaplasia, 19 had moderate anaplasia, and 16 had severe anaplasia. All tumors had gain of 1q, 18 tumors had gain of 6p, 6 tumors had gain of 9q, and 36 tumors had loss of 16q. Fourteen tumors (33%) had at least 1 histologic high-risk feature. Distant metastases developed in 4 patients (10%), and 2 patients demonstrated tumor growth past the cut end of the optic nerve. Two patients (5%) demonstrated second primary tumors after enucleation, and 2 patients (5%) died of metastatic retinoblastoma. Median follow-up for the survivors was 15 years (SD, 5 years; Table 2).

**Table 2:** Basic Clinical Characteristics of Included Patients

Variable	Data
No.	42
Age at enucleation	1.4 ± 0.9
Laterality	
Unilateral	29 (69)
Bilateral	13 (31)
Anaplasia grade	
Mild	7 (17)
Moderate	19 (45)
Severe	16 (38)
Chromosomal alterations	
Gain of 1q	42 (100)
Gain of 6p	18 (43)
Gain of 9q	6 (14)
Loss of 16q	36 (86)
Secondary tumors	2 (5)
Metastases	4 (10)

Data are presented as no. (%) or mean ± standard deviation.

### Digital Cell Morphometry

The average number of cells measured in each tumor was 5653 (SD, 1545). We found no statistically significant differences between pathologists’ classifications of nonsevere anaplasia (mild plus moderate) versus severe anaplasia in morphometric variables related to measurements of size and shape of tumor cell nuclei and cytoplasm (*P* > 0.05, Mann–Whitney *U* test or Student *t* test).

**Figure 2:**
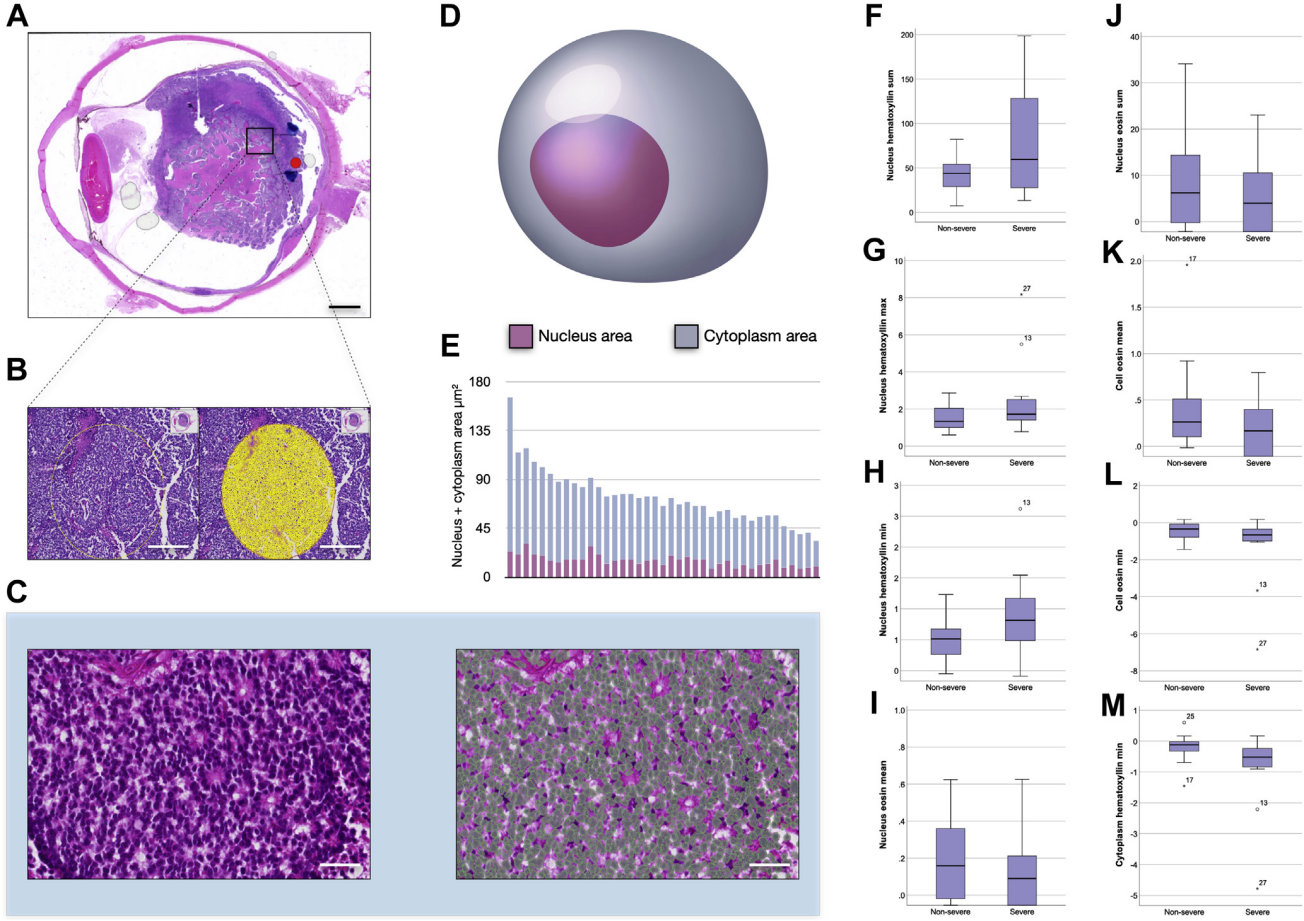
Cell morphometric analysis. **A**, Diagram showing primarily endophytic retinoblastoma in an enucleated eye. **B**, Photomicrograph showing, in one area of this tumor, a circular region of interest (yellow circle). **C**, Photomicrographs showing, within this region of interest, morphometric parameters of all cells are measured with digital image analysis software. On the right, in higher magnification of a tumor area with Flexner-Wintersteiner rosettes, the software has identified the size, shape, and staining features of tumor nuclei. Green polygons have been added to illustrate the outlines of some of the individual cells. **D**, Illustration showing a tumor cell with the nucleus area and cytoplasm area marked in purple and steel blue, respectively. **E**, Stacked bar graph showing nucleus (purple) plus cytoplasm area (steel blue) in the analyzed tumors. **F**, Box-and-whisker plot showing the sum of hematoxylin staining density in nuclei from tumors with nonsevere anaplasia (left box) versus severe anaplasia (right box). **G**, Box-and-whisker plot showing the maximum hematoxylin staining density in nuclei. **H**, Box-and-whisker plot showing the minimum hematoxylin staining density in nuclei. **I**, Box-and-whisker plot showing the mean eosin staining density in nuclei. **J**, Box-and-whisker plot showing the sum of eosin staining density in nuclei. **K**, Box-and-whisker plot showing the mean eosin staining density in cells (nucleus plus cytoplasm). **L**, Box-and-whisker plot showing the minimum eosin staining density in nuclei. **M**, Box-and-whisker plot showing the minimum hematoxylin staining density in cytoplasms. All significant on a 0.05 level. ° = Outlier. Scale bars: (**A**) 2 mm, (**B**) 200 μm, and (**C**) 75 μm.

However, several of the 15 variables relating to staining characteristics differed significantly. Generally, the hematoxylin staining intensity was significantly greater and eosin staining intensity was significantly less in tumors with severe anaplasia. The sum of hematoxylin staining intensity in tumor cell nuclei was significantly higher in tumors with severe anaplasia (*P* = 0.008). Similarly, the maximum (*P* = 0.036) and minimum (*P* = 0.045) hematoxylin nuclear staining intensities were significantly higher in tumor cell nuclei in tumors with severe anaplasia. However, the mean of nuclear eosin staining intensity (*P* = 0.022) and sum of eosin staining intensity (*P* = 0.014) were significantly lower in tumors with severe anaplasia. In entire cells (nucleus plus cytoplasm), the mean and minimum eosin staining intensities were significantly lower in tumors with severe anaplasia (*P* = 0.039 and *P* = 0.036, respectively). In cytoplasms only, the minimum hematoxylin staining intensity was significantly lower in tumors with severe anaplasia (*P* = 0.007; Table 3; Fig 2).

**Table 3:** Distribution of Morphometric Variables across Degrees of Anaplasia

Variable	Nonsevere Anaplasia	Severe Anaplasia	*P* Value
No.	26	16	
Nucleus			
Area	7.456 ± 2.402	8.603 ± 5.158	0.722
Perimeter	11.501 ± 1.89	12.370 ± 3.833	0.352
Circularity	0.705 ± 0.077	0.668 ± 0.089	0.205
Caliper			
Maximum	4.526 ± 0.792	4.970 ± 1.549	0.247
Minimum	2.457 ± 80.388	2.437 ± 0.825	0.918
Eccentricity	0.788 ± 0.073	0.840 ± 0.109	0.089
Hematoxylin OD			
Mean	0.920 ± 0.469	1.624 ± 1.226	0.081
Sum	42.074 ± 20.885	81.348 ± 64.897	**0.008**
SD	0.263 ± 0.166	0.387 ± 0.428	0.327
Maximum	1.516 ± 0.645	2.516 ± 2.165	**0.036**
Minimum	0.401 ± 0.545	0.892 ± 0.724	**0.045**
Range	1.115 ± 0.724	1.623 ± 1.699	0.297
Eosin OD			
Mean	0.202 ± 0.299	0.319 ± 1.042	**0.022**
Sum	8.590 ± 12.297	13.667 ± 40.728	**0.014**
SD	0.243 ± 0.171	0.369 ± 0.398	0.207
Maximum	0.782 ± 0.568	0.533 ± 0.622	0.230
Minimum	0.333 ± 0.44	1.095 ± 1.9	0.058
Range	1.116 ± 0.783	1.628 ± 1.594	0.297
Cell			
Area	32.352 ± 14.49	27.632 ± 10.956	0.327
Perimeter	22.984 ± 5.161	21.474 ± 4.324	0.385
Circularity	0.647 ± 0.139	0.565 ± 0.164	0.119
Caliper			
Maximum	8.976 ± 1.883	8.641 ± 1.881	0.613
Minimum	4.791 ± 1.327	4.107 ± 0.99	0.120
Eccentricity	0.803 ± 0.062	0.838 ± 0.096	0.194
Eosin OD			
Mean	0.374 ± 0.417	0.017 ± 0.596	**0.039**
SD	0.291 ± 0.243	0.487 ± 0.661	0.174
Maximum	1.306 ± 1.314	1.331 ± 1.564	0.792
Minimum	0.438 ± 0.474	1.325 ± 1.984	**0.036**
Cytoplasm hematoxylin OD			
Mean	0.444 ± 0.469	0.186 ± 0.496	0.130
SD	0.254 ± 0.231	0.403 ± 0.504	0.137
Maximum	1.250 ± 1.311	1.292 ± 1.574	0.865
Minimum	0.193 ± 0.374	0.940 ± 1.346	**0.007**
Nucleus-to-cell area ratio	0.295 ± 0.159	0.350 ± 0.201	0.364

OD = optical density; SD = standard deviation.

Data are presented as mean ± standard deviation, unless otherwise indicated. Boldface indicates statistical significance.

### Fluorescence In Situ Hybridization

**Figure 3:**
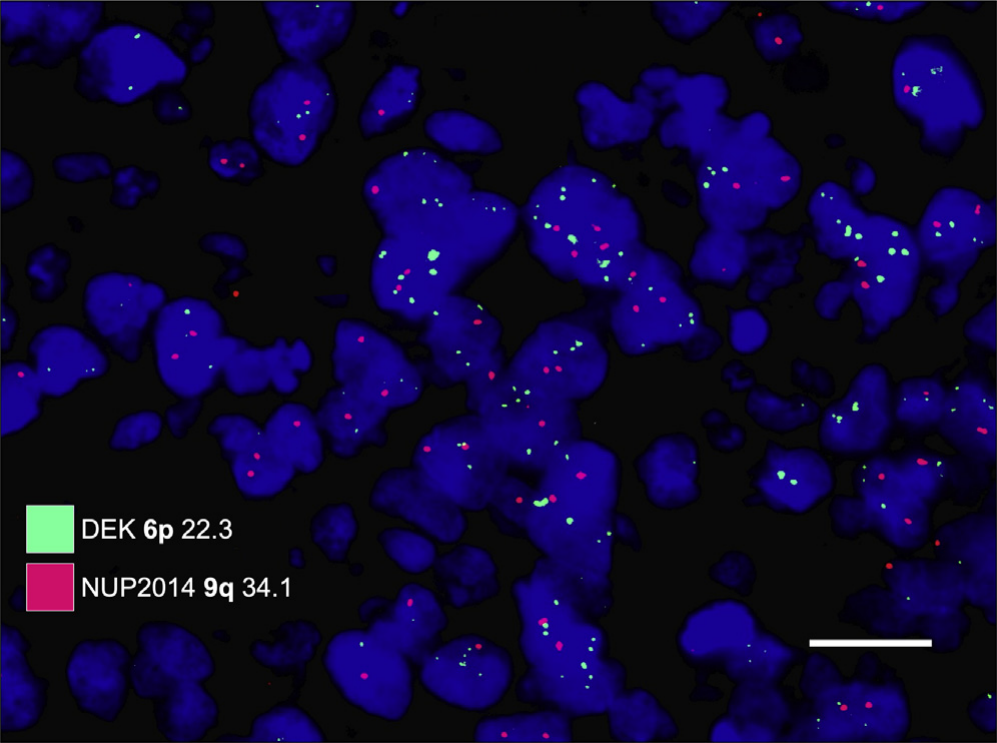
Examples of fluorescence in situ hybridization with probes for 6p (green) and 9q (red). This tumor shows increased numbers of green signals, corresponding to gain of 6p. White scale bar = 20 μm.

All 16 tumors with severe anaplasia had gain of 6p (Fig 3). Furthermore, 2 tumors with moderate anaplasia also had gain of 6p, but no tumor with mild anaplasia. Tumors with severe anaplasia were significantly more likely to harbor 6p gains than tumors with nonsevere anaplasia (*P* < 0.001, Fisher exact test). However, severe anaplasia was not correlated to gain of 9q (*P* = 0.66, Table 4). The relative risk for severe anaplasia in tumors with 6p gain was 15.0 (95% confidence interval, 1.0–220.9; *P* = 0.05).

**Table 4:** Degree of Anaplasia versus Chromosomal Alterations

	Degree of Anaplasia, No. (%)		*P* Value
	Nonsevere (n = 26)	Severe (n = 16)	
Gain of 6p	2 (8)	16 (100)	<0.001
Gain of 9q	3 (12)	3 (19)	0.66

### Histologic High-Risk Features

Of the 14 tumors with at least 1 histological high-risk feature, 8 (57%) had gain of 6p. Tumors with histologic high-risk features were not significantly more likely to harbor 6p gains (*P* = 0.10, Fisher exact test) or 9q gains (*P* = 0.60, Fisher exact test) or to have severe anaplasia (*P* = 0.21, Fisher exact test) than tumors without histologic high-risk features (Table 5).

**Table 5:** Histologic High-Risk Features versus Anaplasia and Chromosomal Aberrations

	No. of Histologic High-Risk Features, No. (%)		*P* Value
	0 (n = 18)	≥1 (n = 10)	
Severe anaplasia	4 (22)	5 (50)	0.21
Gain of 6p	4 (40)	6 (60)	0.10
Gain of 9q	2 (7)	2 (20)	0.60

### Whole Genome Sequencing

**Figure 4:**
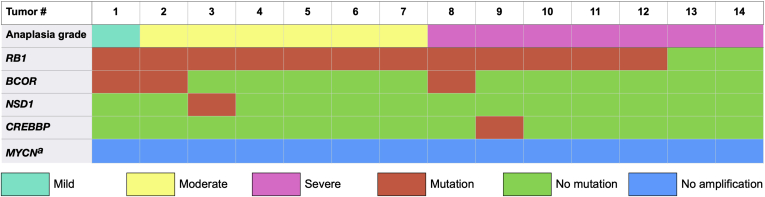
Heat map showing the distribution of *RB1*, *BCOR*, *NSD1*, and *CREBBP* mutations and *MYCN* amplifications over 14 tumors with mild to severe anaplasia. ^a^Gene amplification.

Severe anaplasia did not correlate significantly to *CREBBP*, *NSD1*, *BCOR*, or *RB1* mutations (*P* > 0.5). No tumor showed *MYCN* amplification (Table 6; Fig 4).

**Table 6:** Degree of Anaplasia versus Genetic Aberrations

	Degree of Anaplasia, No. (%)		*P* Value
	Nonsevere (n = 7)	Severe (n = 7)	
*RB1* mutation	7 (100)	5 (71)	0.5
*BCOR* mutation	2 (29)	1 (14)	1.0
*NSD1* mutation	1 (14)	0 (0)	1.0
*CREBBP* mutation	0 (0)	1 (14)	1.0
*MYCN* amplification	0 (0)	0 (0)	1.0

### Survival

Of the six patients who demonstrated distant metastases or had tumor growth past the cut end of the optic nerve, 5 harbored gain of 6p (*P* = 0.07, Fisher exact test). The relative risk for extraocular spread in tumors with 6p trended toward significance at 6.7 (95% confidence interval, 0.9–52.2; *P* = 0.07).

**Figure 5:**
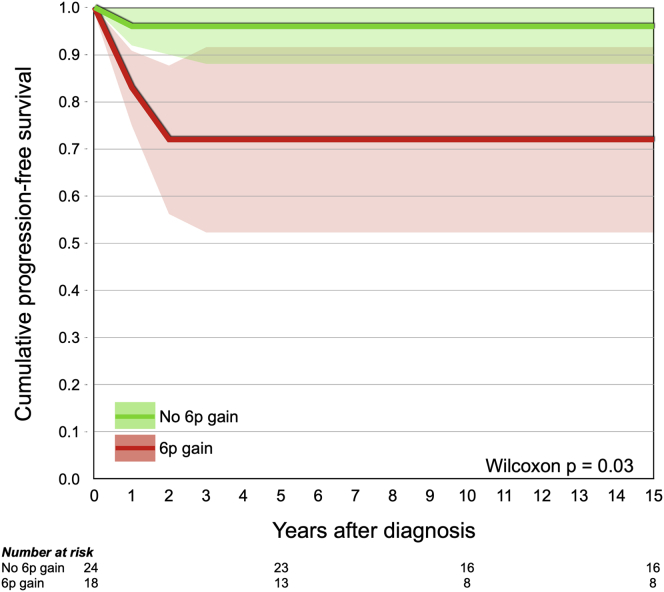
Survival curve showing cumulative progression-free survival for patients with retinoblastoma with (red) and without (green) gain of chromosome 6p. Tumor progression was defined as presence of distant or local metastases or tumor growth beyond the cut end of the optic nerve at the time of enucleation or later.

Neither severe anaplasia (*P* = 0.57, Fisher exact test), gain of 9q (*P* = 0.48), nor histologic high-risk features (*P* = 0.60) correlated to extraocular spread. In cumulative incidence analysis, patients with gain of 6p showed significantly shorter progression-free survival (*P* = 0.03, Wilcoxon test; Fig 5). Patients with 6p gain also showed a trend for shorter disease-specific survival (cumulative proportion of patients not dead of retinoblastoma), although this was not significant on the 0.05 level (*P* = 0.07). Patients with severe anaplasia did not show shorter disease-specific survival (*P* = 0.56) or progression-free survival (*P* = 0.11). Because the cause of death of both patients who died was metastatic retinoblastoma and no other causes of death was present, the disease-specific survival was identical to overall survival.

Three retinoblastomas showed a concurrent retinocytoma in the same eye. The presence of a retinocytoma was not correlated to metastasis, histologic high-risk features, gain of 9q, gain of 6p, or severe anaplasia (*P* > 0.2).

## Discussion

In this study, the degree of anaplasia in retinoblastoma was correlated to chromosome 6p gains and changes to tumor cell size and staining characteristics. Hematoxylin staining intensities were significantly higher and eosin staining intensities were significantly lower in severely anaplastic tumor cells. This could be explained by a higher content of phosphorylated chromatin, RNA, ribosomes, and rough endoplasmic reticulum in severely anaplastic cells. Severe anaplasia has been identified as a useful histopathologic criteria in identifying patients with an increased risk of metastasis with potential need for adjuvant therapy in patients with retinoblastoma who may not exhibit high-risk histologic features.^10^ For more than a century, pathologists have sought to describe morphologic changes to separate benign from malignant cells and to determine the aggressiveness of the latter. Characteristics such as larger nuclei, nuclear membrane irregularities, abnormal chromatin distribution, increased nuclear-to-cytoplasmic ratios, and hyperchromasia are typically associated with malignancy, but are hard to quantify. Digital morphology is therefore a long-awaited tool to reduce subjectivity in these assessments. With the rapidly growing number of digital image analysis and artificial intelligence solutions for assisting pathologists with diagnosis, treatment prediction, and prognostication, further developments in this field should be expected.^37^, ^38^, ^39^, ^40^, ^41^

Further, severe anaplasia correlated strongly with gain of chromosome 6p. The central part of the short arm of chromosome 6p has been reported to harbor oncogenes that are linked to tumor progression, and gains at 6p have been associated with metastatic disease and poor prognosis in other cancers.^14^ The p arm of chromosome 6 harbors the codes of multiple genes including nuclear protein 7 (*NOL7*).^42^ Loss of *NOL7* is associated with retinoblastoma, and reintroduction of *NOL7* suppresses in vivo tumor growth of cervical cancers.^43^ *NOL7* has been shown to function within the nucleus and nucleolus, and inhibition of *NOL7* may lead to abnormal changes to the nucleus and nucleolus, and therefore may contribute to the hyperchromasia observed in our study.^44^ All included tumors with 6p gain had moderate to severe nuclear anaplasia with higher levels of necrosis, focal invasion of intraocular structures, and higher levels of seeding, whereas tumors that did not have 6p gain demonstrated features ranging from mild to moderate anaplasia. The strong association among 6p gain, focal invasion, and histologic high-risk factors confirms findings in previous publications.^22^ Further, the prognostic importance of histologic high-risk factors, 6p gains, and degree of anaplasia has been shown repeatedly, whereas some debate remains regarding the importance of focal invasion.^22^^,^^45^, ^46^, ^47^

Therefore, gain of 6p could be used as a prognostic biomarker. The information on 6p status may be added to other factors when making treatment decisions, for example, IIRC or AJCC classification and assessment of histologic high-risk factors. Considering that the IIRC classification is predictive of treatment success in half of cases, 6p status may be used for further risk stratification and to guide decisions to enucleate in selected patients. This role is most anticipated for intermediate tumors where the right course of action is least obvious. Patients with small, clearly demarcated tumors without seeding and with no signs of invasion of the anterior segment, choroid, or optic nerve likely should not be treated more aggressively, even if a 6p gain has been detected. Similarly, large diffuse tumors with signs of invasion should be enucleated even if no 6p gain is detected. Between these extremes, where the risk for hesitation and indecisiveness on the appropriate course of treatment is highest, lies the natural place for 6p ploidy analysis. Previous research found that 6p gains predict enucleations in IIRC group D and AJCC cT2b eyes, whereas samples have been too small for statistical significance in IIRC group E and AJCC cT3 eyes.^7^ Because evaluations based on aqueous humor and tumor tissue in enucleated eyes are highly concordant, it may be recommended that clinicians in such intermediate situations sample aqueous humor and opt for a more aggressive line of treatment if a 6p gain is detected.^7^ Similarly, detection of a 6p gain in tumor tissue obtained from an enucleated eye is likely to lead to increased vigilance and metastatic screening. However, to the best of our knowledge, neither 6p gains nor degree of anaplasia are independent predictors of metastasis or death when adjusting for high-risk histopathologic features.^7^^,^^10^^,^^22^^,^^48^

This study has several limitations. A relatively limited number of tumors were included, and it is possible that we would have found significant correlations with histologic high-risk features, anaplasia, and decreased survival in a larger cohort.^10^ An *RB1* mutation was not found in all tumors, presumably because of complex structural variants, regulatory region alterations, or similar, rather than an actual lack of mutation. Although largely automatized without risk of human errors and variability, significant steps in our digital image analysis still required manual input, not least the definition of regions that were representative of a tumor’s degree of anaplasia. The selection of these regions was made by ophthalmic pathologists. Theoretically, this could have influenced the results, although previous examinations of this method have indicated high interobserver concordance.^18^

In conclusion, gain of chromosome 6p correlates with severe anaplasia and extraocular spread of retinoblastoma. The degree of anaplasia is quantifiable by digital morphometry of tumor cell staining characteristics after enucleation. Analysis of chromosome 6p polyploidy via either direct or liquid biopsy may be valuable in prognostication of this disease.
